# ‘We had to climb mountains and cross fast-flowing rivers’

**DOI:** 10.1098/rstb.2008.4012

**Published:** 2008-11-27

**Authors:** Taka Gomea

**Affiliations:** Port Moresby, c/o Papua New Guinea Institute of Medical ResearchPO Box 60, Goroka, EHP 441, Papua New Guinea

It was Dr Vincent Zigas who first started research into kuru and Dr Carleton Gajdusek joined him later. Vincent would drop us off and pick us up with his Landrover and we would patrol through the kuru-affected region with Carleton. Vincent was from Europe and we had great difficulty in understanding his Tok Pisin. He knew this, so he would purposely talk in a way that made us laugh. He was a good-natured man and was always reminding us to take care of Carleton when we were patrolling.

When we patrolled with Carleton it was under difficult conditions; we had to climb mountains and cross fast-flowing rivers. When we approached some villages they tried to chase us away, threatening us with their bows and arrows. We would placate them by giving them salt and other small presents.

The longest patrol that I took part in was to Menyamya via Agakamatasa, Dunkwi, Simbari and Mt Yelia. From Menyamya we took a plane to Lae, where Carleton left to go to America. After cleaning ourselves, Anua, Masasa, Tiu, Tose, Haus Kapa and I boarded a Dakota cargo plane bound for Kainantu. When we were about to land in Kainantu the plane's cargo doors flew open and I went and told the plane's crew. We all held on to the co-pilot so he could lean out of the plane and grab the doors and close them. When we landed the pilot told us that if we had not closed the doors we would have all died. Then we walked back to Okapa aided by a kerosene lamp.

Jack Baker sent me to Goroka to train as an aid post orderly and afterwards I became an ambulance driver at Port Moresby General Hospital. In Moresby ‘the man from the mountains’ married a girl from the coast and we have lived happily ever since.

Finally, I would just like to say that I really wanted kuru to stop and that is why I was happy to work so hard.

